# Lectin domains at the frontiers of plant defense

**DOI:** 10.3389/fpls.2014.00397

**Published:** 2014-08-13

**Authors:** Nausicaä Lannoo, Els J. M. Van Damme

**Affiliations:** Laboratory of Biochemistry and Glycobiology, Department of Molecular Biotechnology, Ghent UniversityGhent, Belgium

**Keywords:** carbohydrate, innate immunity, lectin, protein–carbohydrate interaction, PRR

## Abstract

Plants are under constant attack from pathogens and herbivorous insects. To protect and defend themselves, plants evolved a multi-layered surveillance system, known as the innate immune system. Plants sense their encounters upon perception of conserved microbial structures and damage-associated patterns using cell-surface and intracellular immune receptors. Plant lectins and proteins with one or more lectin domains represent a major part of these receptors. The whole group of plant lectins comprises an elaborate collection of proteins capable of recognizing and interacting with specific carbohydrate structures, either originating from the invading organisms or from damaged plant cell wall structures. Due to the vast diversity in protein structures, carbohydrate recognition domains and glycan binding specificities, plant lectins constitute a very diverse protein superfamily. In the last decade, new types of nucleocytoplasmic plant lectins have been identified and characterized, in particular lectins expressed inside the nucleus and the cytoplasm of plant cells often as part of a specific plant response upon exposure to different stress factors or changing environmental conditions. In this review, we provide an overview on plant lectin motifs used in the constant battle against pathogens and predators during plant defenses.

## INTRODUCTION

In nature, plants are constantly exposed to a plethora of different pathogens including bacteria, viruses, and fungi. Whereas the interaction with some of these organisms can be beneficial, most microbial infection is harmful for the plant (Dangl et al., 2013). In order to resist pathogen colonization, plants developed a highly sophisticated, multilayered system enabling the plant to recognize invading pathogens and mount rapidly efficient defense responses (Muthamilarasan and Prasad, 2013; Wirthmueller et al., 2013).

Microbial entry into the host tissue is a critical step in causing infection. Pathogens can enter plants through natural openings such as stomata, hydathodes, lateral roots, or through accidental wounds, but can also form specialized structures such as haustoria to penetrate directly into the plant surface (Melotto et al., 2006; Gudesblat et al., 2009). Many phytopathogens also produce lytic enzymes to damage the plant cell wall in favor of pathogen invasion. Perception of the invading pathogen is the first step in the plant’s defense and is governed by cell surface transmembrane receptors. These pattern recognition receptors or PRRs are able to recognize two types of molecules.

The first group encompasses the damage-associated molecular patterns (DAMPs) which are produced in the plant apoplast as a consequence of pathogen entry. Examples include cell wall fragments such as oligogalacturonides and cellulose fragments, cutin monomers, and peptides such as systemin, defensins, and phytosulfokines (Ryan, 2000; Nühse, 2012; Albert, 2013). PRRs are also able to recognize conserved microbial structures, known as pathogen- or microbe-associated molecular patterns (PAMPs/MAMPs), which are essential for the microbial physiology and the pathogen’s fitness (Newman et al., 2013; Wirthmueller et al., 2013). Examples of PAMPs/MAMPs include lipopolysaccharides (LPS) of Gram-negative bacteria, peptidoglycan (PGN) of Gram-positive bacteria, bacterial flagellins, eubacterial elongation factors (EF-Tu), and fungal cell wall derived glucans, chitins, and proteins.

Upon PAMP/MAMP and DAMP perception by the PRRs, the so-called PAMP/MAMP-triggered immunity (PTI/MTI) response is activated which gives rise to downstream intracellular signaling events such as activation of mitogen-activated protein kinases, production of reactive oxygen species and transcriptional reprogramming ultimately leading to a complex output response of the plant that limits microbial growth (Wirthmueller et al., 2013).

However, successful pathogens have elaborated a counter defense response to overcome PTI by means of expression of specific elicitors or effector proteins [also known as avirulence (Avr) proteins; Grant et al., 2006]. Pathogenic bacteria typically inject these effectors directly into the cytoplasm of the plant host cell through type III secretion mechanisms to suppress and/or block PRR-dependent signaling, to facilitate nutrient acquisition and to contribute to the pathogen’s dispersal which can lead to effector-triggered susceptibility (ETS; Block et al., 2013; Cui et al., 2013).

As a counter move, plants have co-evolved a second layer of defense, known as effector-triggered immunity (ETI) which, in contrast to PTI/MTI acts mostly inside the plant cell. In ETI, specific resistance (*R*) genes become expressed upon recognition of an effector to produce defense proteins. The majority of the R proteins include nucleotide-binding leucine-rich repeat (NB-LRR)-containing proteins. The outcome of PTI/MTI and ETI can lead to programmed cell death of the host cell via (local) activation of a hypersensitive response (HR), but can also initiate systemic acquired resistance (SAR) to activate defenses in distal, non-infected parts of plants in order to establish a heightened state of immunity throughout the plant (Thomma et al., 2011).

## PATHOGEN RECOGNITION RECEPTORS (PRRs)

**FIGURE 1:**
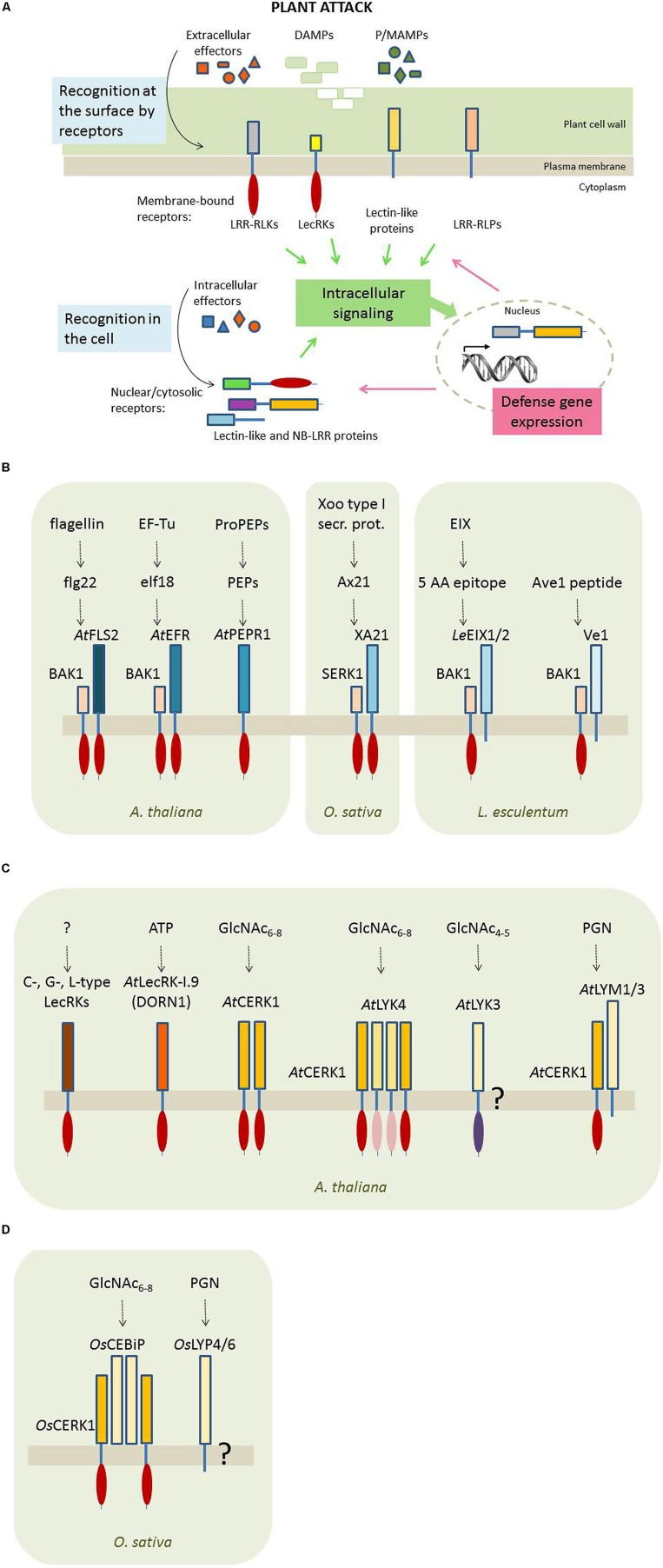
**Plant innate immunity. (A)** Perception of pathogen/microbe-associated molecular patterns (P/MAMPs), damage-associated molecular patterns (DAMPs) and pathogen-derived effector proteins. Plants sense P/MAMPs, DAMPs, and effectors through membrane-bound and intracellular (soluble) receptors. Four types of membrane-bound receptors can be distinguished: the LRR-type receptor kinases (LRR-RLKs) and proteins (LRR-RLPs), and the receptor kinases and proteins with lectin domains (called LecRKs and lectin-like proteins, respectively). Soluble receptors known thus far include NB-LRR proteins as well as nucleocytoplasmic lectins. Upon ‘danger’ perception, these receptors trigger intracellular signals which ultimately will result in altered expression of defense-related genes. Legend: ellipses represent kinase domains; bars represent other protein motifs, including LRRs and lectin domains. **(B)** Transmembrane PRRs detect P/MAMPs through protein–protein interactions. Bars represent LRR domains, red ellipses indicate functional kinase domains. **(C)** Transmembrane PRRs with lectin domains identified in *Arabidopsis thaliana*. In the case of the LysM domain evidence supports protein–carbohydrate interactions to detect P/M/DAMPs. Bars represent lectin domains, including C-, G-, and L-type (brown) and LysM (yellow) domains. Ellipses represent kinase domains (red = functional, pink = non-functional, purple = putative kinase domain). **(D)** Transmembrane PRRs with lectin domains identified in *Oryza sativa.* The LysM domain recognizes P/M/DAMPs through specific binding of chitin fragments. Bars represent the LysM lectin domains; ellipses represent functional kinase domains.

The cell wall confers the first tier of the plant’s immunity (Malinovsky et al., 2014). The extracellular PRRs are able to detect pathogen determinants (the so-called PAMPs/MAMPs), DAMPs and effectors at the surface of the plant cell and are used to translocate the extracellular message of ‘danger’ to the intracellular environment to trigger appropriate defense mechanisms (**Figure 1A**; Dubery et al., 2012). The PRR family encompasses two groups of plasma membrane-localized proteins: the receptor-like kinases (RLKs) and the receptor-like proteins (RLPs). RLKs are single-pass transmembrane proteins with an extracellular domain that is responsible for the perception of the P/M/DAMPs and an intracellular serine/threonine kinase domain that activates the downstream signaling responses. RLPs possess a similar structure but, because they only have a short cytosolic domain without an obvious signaling module, they depend on the association with kinases for signaling. However, there is emerging evidence that upon ligand binding RLKs also form homodimers or heterodimers with other kinases and RLPs and as such function in multiprotein complexes to initiate plant immunity (Boller and Felix, 2009; Böhm et al., 2014; Han et al., 2014; Macho and Zipfel, 2014).

Thus far, only a limited number of RLKs and RLPs that may function in plant immunity have been functionally characterized. Matching these proteins to their ligands is still a challenging study. The majority of the known PRR ectodomains contains LRRs for direct/indirect recognition of pathogenic effector proteins (**Table 1**). In addition, a large diversity of membrane-bound and soluble PRRs have been described to carry lectin domains that are implicated in the recognition of carbohydrate structures from microbial organisms or derived from plant cell wall damage (**Tables 2** and **3**).

**Table 1:** LRR-type PRRs involved in plant defense signaling.

PRR	Plant species	Ligand	Reference
**LRR-RLK type**
*At*FLS2	*Arabidopsis thaliana*	Flagellin (Flg22)	Chinchilla et al. (2006)
*At*EFR	*A. thaliana*	Ef-TU	Zipfel et al. (2006)
XA21	*Oryza sativa*	Activator of XA21 (Ax21)	Lee et al. (2009)
XA3/XA26		Epitope derived from *Xanthomonas oryzae* pv *oryzae*	Sun et al. (2004)
SR160	*Lycopersicon peruvianum*	(pro)systemin	Scheer and Ryan (2002)
PEPR1	*A. thaliana*	PEPR1	Krol et al. (2010)
NORK	*Medicago truncatula*	?	Endre et al. (2002)
SYMRK	*Lotus japonicus*	?	Stracke et al. (2002)
**LRR-RLP type**
LeEIX2	*Lycopersicon esculentum*	Xylanase (EIX)	Ron and Avni (2004)
Ve1	*L. esculentum*	Ave1 peptide	Fradin et al. (2009)

**Table 2:** Membrane-bound lectin-type PRRs involved in plant defense signaling and symbiosis.

PRR	Plant species	Ligand	Reference
**LysM-RLK type**
AtCERK1	*Arabidopsis thaliana*	Chitin	Miya et al. (2007), Brotman et al. (2012)
AtLYK3	*A. thaliana*	Chitin	Paparella et al. (2014)
AtLYK4	*A. thaliana*	Chitin	Wan et al. (2012)
OsCERK1	*Oryza sativa*	Chitin (when in combination with OsCEBiP)	Kaku et al. (2006)
NFR1	*Lotus japonicus*	Lipochitooligosaccharide Nod factors	Radutoiu et al. (2003)
LYK3	*Medicago truncatula*	Lipochitooligosaccharide Nod factors	Knogge and Scheel (2006)
NFR5	*L. japonicus*	Lipochitooligosaccharide Nod factors	Radutoiu et al. (2003), Madsen et al. (2003)
SYM10	*Pisum sativum*	Lipochitooligosaccharide Nod factors?	Madsen et al. (2003)
LYK4	*M. truncatula*	Lipochitooligosaccharide Nod factors	Limpens et al. (2003)
NFP	*M. truncatula*	Lipochitooligosaccharide Nod factors	Mulder et al. (2006)
**LysM-RLP type**
LYM1/AtLYP2, LYM3/AtLYP3	*A. thaliana*	Peptidoglycan	Willmann et al. (2011), Tanaka et al. (2013)
OsCEBiP	*O. sativa*	Chitin	Shimizu et al. (2010)
OsLYP4, OsLYP6	*O. sativa*	Chitin + Peptidoglycan	Liu et al. (2012a)

**Table 3:** Nucleocytoplasmic lectin domains involved in plant defense signaling.

Lectin domain	Carbohydrate Specificity	Subcellular localisation	Examples
Amaranthin domain	GalNAc, T-antigen	nucleus, cytosol	Amaranthin, Hfr-2
EUL domain	Galactosides, high-mannose *N*-glycans	nucleus, cytosol	EEA, ArathEULS3
Jacalin domain	Mannose-specific subgroup / galactose-specific subgroup	nucleus, cytosol / vacuole	Orysata, TaVER2, TaHfr-1,TaJA-1
Nictaba domain	(GlcNAc)_n_, high-mannose *N*-glycans, complex *N*-glycans	nucleus, cytosol	Nictaba, PPL
Ricin-B domain	Gal/GalNAc, Siaα2,6Gal/GalNAc	Vacuole, nucleus, cytosol	Ricin, abrin, SNA-I, SNA-V

## PATHOGEN RECOGNITION BASED ON PROTEIN–PROTEIN INTERACTIONS

The study of plant–pathogen interactions has focused on those PRRs which use protein–protein interactions to recognize invading pathogens. Phytopathogens are recognized upon perception of characteristic epitopes present on their surface and essential for the pathogen’s survival. These epitopes are mostly recognized by plant cell surface receptors carrying LRR motifs in their ectodomain structures and a kinase domain in their intracellular domain, collectively named the LRR-RLKs. Since these protein–protein interactions have been the subject of several recent overview papers, we only briefly summarize some plant LRR-RLKs and LRR-RLPs (**Figure 1B** and **Table 1**).

Amongst the plant PRRs of the LRR-RLK type, the *Arabidopsis* LRR-RLK *At*FLS2 (Flagellin Sensing 2) is the best-studied protein, containing 28 extracellular LRRs. This FLS2 recognizes bacterial flagellin via perception of the conserved 22-amino acid epitope flg22. *At*FLS2 directly interacts with flg22 resulting in phosphorylation of *At*FLS2 and immediate dimerization with its co-receptor BAK1/SERK, another LRR-RLK. Transphosphorylation of the kinase domain of BAK1 enables conformational changes and subsequent release of phosphorylated BAK1 leading to activation of downstream MAPK defense signaling (Gómez-Gómez et al., 2001; Chinchilla et al., 2006, 2007; Schulze et al., 2010). In the absence of PAMP recognition, BAK1 itself interacts with the pseudokinase BIR2 (also LRR-RLK-type) to prevent FLS2-BAK1 heterodimerization (Halter et al., 2014). After flg22 perception, *At*FLS2 is subject to endocytosis and degradation by the E3 ubiquitin ligase PUB12/13 to prevent continuous defense signaling. Newly synthesized *At*FLS2 is incorporated in the plasma membrane at later times (Smith et al., 2014). In turn, virulent *Pseudomonas syringae* pathovars produce effector proteins, such as AvrPto, AvrPtoB, and AvrPphB to destabilize *At*FLS2 and thus compromise host immunity (Block and Alfano, 2011).

The transmembrane protein *At*EFR represents another Arabidopsis LRR-RLK-type receptor involved in bacterial PAMP signaling (Zipfel et al., 2006). The ectodomain of *At*EFR consists of 24 LRRs and is involved in the perception of the elf18 peptide, a conserved N-terminal fragment of bacterial elongation factor Tu. Many of the signaling compounds downstream of *At*EFR are shared with *At*FLS2, and *At*EFR also requires dimerization with *At*BAK1 for signaling. However, the action of *At*EFR is independent of flagellin perception and unlike *At*FLS2, *At*EFR requires N-glycosylation to become functional. Indeed, a single *N*-glycan is crucial for receptor abundance and ligand recognition between the pathogen and the plant cell surface (Häweker et al., 2010).

Rice plants use the transmembrane XA21 receptor kinase to confer immunity toward a number of *Xanthomonas oryzae* pv *oryzae* (*Xoo*) isolates, which cause leaf blight in rice. The XA21 receptor recognizes Ax21, a sulfated 17-amino acid peptide derived from the *Xoo* type I secreted protein (Lee et al., 2009). Also here, the action of XA21 is tightly regulated. Without PAMP, XA21 is kept in an inactive state through binding with and autophosphorylation by the ATPase XB24. Upon binding of Ax21 to XA21, the XB24/XA21 dimer dissociates and the XA21 kinase domain is released and translocated to the cell nucleus for subsequent immune signaling (Park and Ronald, 2012). Chen et al. (2014) recently reported that XA21 can also be found in a constitutive heteromeric complex with a BAK1 ortholog, named OsSERK2, and undergoes bidirectional transphosphorylation to confer resistance to the *Xanthomonas* bacterium.

Tomato plants encode several cell-surface LRR-RLPs such as LeEIX1/EIX2 and Ve1 which confer resistance toward *Trichoderma* and race 1 strains of *Verticillium* pathogens, respectively (Ron and Avni, 2004; Fradin et al., 2009). The ethylene-inducing xylanase EIX is a fungal β-1–4-endoxylanase that is used by *Trichoderma viride* to enter tomato and tobacco plants. The epitope that is recognized by the plants to elicit defense responses constitutes five amino acids that are not involved in the enzymatic activity (Rotblat et al., 2002). Both LeEIX1/EIX2 can bind EIX, but only LeEIX2 transmits the signal to activate immune responses (Ron and Avni, 2004). The ligand of the Ve1 receptor is the Ave1 peptide, a peptide conserved in several fungi and phytopathogenic bacteria. BAK1 signaling is involved in induced defense responses for both LeEIX1 and Ve1 (Fradin et al., 2009; Bar et al., 2010).

## PATHOGEN RECOGNITION BASED ON PROTEIN–CARBOHYDRATE INTERACTIONS

### THE CARBOHYDRATES

Major part of the P/M/DAMPs that are perceived in the plant as ‘danger’ molecules include carbohydrate structures which are either present at the cell surface of the invading pathogen or originate from the plant itself, when released from cell wall degradation caused by pathogen entry. These structures comprise bacterial LPS and PGNs and fungal chitin molecules as well as plant-derived oligogalacturonides and cellulose fragments. Also arabinogalactan proteins residing in the plant cell wall have been reported to be involved in plant immune responses (Newman et al., 2013).

*Lipopolysaccharides* are large outer membrane glycoconjugates found in Gram-negative bacteria that are composed of a lipid, a core oligosaccharide and an *O*-antigen polysaccharide chain. The lipid, called lipid A, is embedded in the bacterial membrane and is linked to the core oligosaccharide by the KDO sugar (3-deoxy-D-mannose-2-octulosonate). The core sugar ends in the *O*-antigen which is composed of oligorhamnans in many phytopathogens.

*Peptidoglycans* are essential cell wall components of Gram-positive and Gram-negative bacteria, and comprise alternating β(1–4) linked *N*-acetylmuramic acid (MurNAc) and *N*-acetylglucosamine (GlcNAc) residues, with a short peptide chain attached to MurNAc.

Chitin is a long-chain polymer of β(1–4) linked GlcNAc residues and is the main component of the fungal cell wall and the exoskeleton of insects. In case of fungi, chitin is cross-linked to β-glucan.

*Oligogalacturonides* are oligomers of α(1,4) linked galacturonosyl residues that are released from plant cell walls upon partial degradation of homogalacturonan (i.e., the major component of pectin) by pathogen attack and also upon mechanical damage.

*Cellulose* is an important component of the plant cell wall, built up of hundreds of β(1–4) linked glucose residues which form long polymer chains. These chains are packed into microfibrils which give strength and flexibility to the plant cell wall.

*Arabinogalactan proteins* are a distinct class of complex, extensively glycosylated hydroxyproline-rich proteins (the so-called proteoglycans), widely distributed among plant species. They consist of a rather small core protein backbone which is *O*-glycosylated by type II arabinogalactan glycans and often contain an N-terminal GPI anchor. AGPs are located near the cell surface, including the plasma membrane, the apoplast, the cell wall, and the intercellular matrix, and have been implicated in many aspects of plant growth and development, such as cell expansion, proliferation, and differentiation. These AGPs are not only involved in establishing a connection between the cell wall and the plasma membrane, but would also extend to the cytoplasm, establishing a continuum between intracellular and extracellular compartments.

### LECTIN DOMAINS INVOLVED IN PLANT IMMUNITY

Lectins are proteins that contain at least one non-catalytic domain which enables them to selectively recognize and bind in a reversible way to specific glycans that are either present in a free form or are part of glycoproteins and glycolipids. Plants express a huge number of highly diverse lectins, exhibiting different molecular structures and binding specificities toward endogenous (plant) glycans as well as to glycans from exogenous (non-plant) origin (Van Damme et al., 2008, 2011).

A lot of plant lectins are constitutively expressed in high amounts in seeds and vegetative storage tissues where they have been shown to play a role in plant defense (Peumans and Van Damme, 1995). In addition, plants also express minute amounts of specific lectins as particular responses toward environmental stresses and pathogen attack. In the absence of plant stress, the inducible lectins are not expressed at detectable levels. Most of the constitutively expressed lectins are synthesized with a signal peptide, and are sequestered in the vacuole or secreted to the extracellular space. In contrast, most of the inducible plant lectins reside in the nucleus and the cytoplasm of a plant cell (Lannoo and Van Damme, 2010).

The majority of the known plant lectins are built up of one or more lectin-like domains coupled to un-related domains such as aerolysin, AIG1, chitinase, dirigent, F-box, Kelch, kinase, LRR, NB-ARC, PAG, or TIR domains (Van Damme et al., 2008). Up till now, most attention of the scientific community dealing with plant innate immunity has been given to transmembrane receptor proteins containing one or more ectopic lectin domains coupled to an intracellular kinase domain. Amongst these lectin receptor kinases (LecRKs), those comprising LysM-type lectin domains are the most studied ones (**Table 2**; Singh and Zimmerli, 2013). However, plants use a broad variety of lectin domains to counteract pathogen attack (**Table 3**).

#### Membrane-bound proteins with a lectin domain

***Lectin receptor kinases (LecRKs).*** Typically, LecRKs are two-domain proteins composed of an N-terminal extracellular lectin domain and a C-terminal cytosolic Ser/Thr kinase domain, separated by a transmembrane region (**Figure 1C**). Based on their lectin domain LecRKs are classified into 4 types; G-, C-, L-, and LysM-type (Singh and Zimmerli, 2013; Vaid et al., 2013). Although these LecRKs consist of at least one domain that shows striking sequence similarity with a lectin motif, very little information is available with respect to the ability of this domain to recognize and interact with specific carbohydratestructures.

*G-type LecRKs* contain an extracellular lectin domain which resembles the *Galanthus nivalis* agglutinin (GNA). However, it remains to be shown whether this sugar binding domain is indeed involved in ligand binding. Based on genome-wide analyses, 32 G-type LecRKs have been identified in *Arabidopsis thaliana* and 100 G-type LecRKs in rice (Vaid et al., 2012). G-type LecRKs function in self-incompatibility reactions in flowering plants (the so-called SRKs) and in plant defense to biotic stress as well as to abiotic stress (Sherman-Broyles et al., 2007; Kim et al., 2009; Sun et al., 2013).

*C-type (calcium-dependent) LecRKs* are mostly found in mammalian proteins that mediate immune responses and play a role in pathogen recognition. In plants, C-type RLKs are rather rare. At present only one C-type LecRK encoding gene has been identified in *A. thaliana* (At1g52310), though its function has not been elucidated yet (Cambi et al., 2005; Bouwmeester and Govers, 2009).

*L-type (legume-like) LecRKs* represent a more abundant group of LecRKs. Thus far, 45 L-type LecRKs have been identified in *A. thaliana*. Based on phylogenetic relationships the genes encoding *Arabidopsis* L-type LecRK can be classified into nine clusters and nine clades (designated with the Roman numerals I to IX). These genes showed variable expression patterns in different tissues and developmental stages in response to stimuli (Bouwmeester and Govers, 2009). Some LecRKs were indeed reported to be involved in plant resistance to pathogens, e.g., *At*LecRK-I.9 is involved in sensing cell wall integrity and defense response to *Phytophthora infestans* (Bouwmeester et al., 2011). *At*LecRK-VI.2 is critical for resistance against *Pseudomonas syringae* and *Pectobacterium carotovorum* (Singh et al., 2012; Huang et al., 2014) while *At*LecRK-IV.3 induces resistance against *Botrytis cinerea* (Huang et al., 2013). Some *At*LecRKs have also been reported to act in hormone signaling (ABA) and stomatal immunity (e.g., *At*LecRK-VI.2 and *At*LecRK-V.5; Singh et al., 2012). L-type LecRKs have also been identified in other plants. For instance, tobacco plants express L-type LecRKs with a role in plant immunity against pathogens and insects (Kanzaki et al., 2008; Gilardoni et al., 2011). In turn, *Medicago* plants contain L-type LecRKs that are involved in symbiosis (Navarro-Gochicoa et al., 2003).

At present, it is not yet clear whether the L-type LecRKs possess lectin activity since the amino acids important for interaction of the legume lectin domain with its specific carbohydrate ligand are poorly conserved. In contrast the hydrophobic site present in the legume-type lectin domain is preserved, suggesting that LecRKs may act in the recognition of small hydrophobic ligands (Huang et al., 2013; Choi et al., 2014). Recently, evidence was obtained that the plasma membrane localized DORN1, encoded by the *AtLecRK-I.9* gene, plays an important role as a receptor for extracellular ATP (**Figure 1C**; Choi et al., 2014). DORN1 lacks the conserved Ca^2+^ and Mn^2+^ binding residues that are critical for carbohydrate binding activity of legume lectins. Early studies also suggested the ability of the legume lectin domain to bind adenine, a component of ATP (Roberts and Goldstein, 1983). However, since adenine was unable to compete with ATP for binding to DORN1, the exact ATP binding site in DORN1 remains to be determined. These data are in good agreement with the fact that extracellular ATP is now perceived as a central signaling molecule in plant stress responses (Cao et al., 2014; Choi et al., 2014).

*LysM LecRKs* are the most studied LecRKs (Figures 1C,D and **Table 2**). They contain ectopic lysin motifs, referred to as LysM domains, which are considered to mediate binding to various types of bacterial PGN and fungal chitin, upon recognition of the GlcNAc moieties (Buist et al., 2008; Gust et al., 2012). The lysine motif, approximately 40 amino acids in length, is a ubiquitous protein domain found in most living organisms except the Archaea. It can be used as a single domain, but is also present in the form of two or occasionally three repeats in a large number of proteins. In most cases LysM motifs are coupled to other protein domains exhibiting some enzymatic activity, such as GlcNAc modification in the case of microbial hydrolases or intracellular signaling for plant kinases.

The *Arabidopsis* chitin elicitor receptor kinase 1 (*At*CERK1, also known as LYK1/RLK1) is the major chitin receptor found in *A. thaliana* (Miya et al., 2007; Petutschnig et al., 2010; Tanaka et al., 2013). It is a membrane-anchored protein with an extracellular domain containing three LysM motifs coupled to an intracellular kinase domain. This kinase domain contains a canonical RD (Arginine-Aspartate) motif in its catalytic loop and possesses autophosphorylation activity, unlike the non-RD kinase domain of typical PRRs. *At*CERK1 was reported to be involved in fungal resistance. The protein directly binds to fungal chitooligosaccharides (GlcNAc_n_ with *n* > 2) through its LysM domains, but only longer oligomers (*n* > 4) trigger immune responses. Liu et al. (2012b) demonstrated that binding of chitin oligomers (*n* = 8) to *At*CERK1 induces homodimerization of the receptor, which is essential for the activation of downstream intracellular signaling, most likely by phosphorylation of both CERK1 cytoplasmic kinase domains.

*At*CERK1 can also mediate perception of PGN when part of a complex with *At*LYM1 and *At*LYM3, two other transmembrane LysM containing proteins lacking an intracellular kinase domain (the so-called LYP proteins; Willmann et al., 2011). Next to *At*CERK1 *A. thaliana* contains four additional LysM RLKs, designated *At*LYK2–5. Since *At*LYK4 and *At*LYK5 can also recognize and bind to chitin molecules but have a non-functional pseudokinase domain it has been suggested they should form a complex with *At*CERK1 to compose a functional chitin receptor (Wan et al., 2012). *At*LYK3 possesses a functional kinase domain, but was suggested be involved in ABA signaling rather than in chitin recognition (Paparella et al., 2014).

Unlike *At*CERK1, the rice ortholog *Os*CERK1 cannot bind directly to chitooligosaccharides. *Os*CERK1, a LysM LecRK with a single extracellular LysM domain, requires heterodimerization with its co-receptor *Os*CEBiP (chitin elicitor binding protein) for chitin binding and subsequent activation of innate immunity (**Figure 1D**). *Os*CEBiP is a transmembrane LysM receptor protein containing three LysM domains and lacking a kinase domain, resembling the *Arabidopsis* proteins *At*LYM1 and *At*LYM3. However, whereas *At*LYM1 and *At*LYM3 are involved in PGN binding through receptor formation with *At*CERK1, *Os*CEBiP seems to play a major role in fungal chitin perception. Upon binding to the fungal GlcNAc_6-8_ oligomers, *Os*CEBiP homo-dimerizes at the plasma membrane of the plant cell. After this ligand-induced dimerization, the *Os*CEBiP sandwich-like structure forms a heteromeric complex with two *Os*CERK1 proteins, to activate intracellular defenses, including ROS signaling, callose deposition, and defense gene expression (Shimizu et al., 2010; Shinya et al., 2012; Hayafune et al., 2014; Kouzai et al., 2014). *Os*LYP4 and *Os*LYP6 are two additional rice LysM proteins lacking a kinase domain which presumably mediate perception of both PGN and chitin, but their action and transmembrane signal transfer remain unclear (Liu et al., 2012a).

Plants not only use LysM LecRKs to recognize pathogenic organisms, they also use them to perceive beneficial organisms such as mycorrhizal fungi and rhizobacteria implicating a dual role of LysM in both innate immunity and symbiosis (**Table 2**; Gust et al., 2012). Examples include the *Lotus japonicus* NRF1 and NRF5 (Radutoiu et al., 2003) and the *Medicago truncatula* LYK3 and LYK4 (Knogge and Scheel, 2006) which can recognize rhizobial lipochitin-oligosaccharide signals or Nod factors.

#### Soluble proteins with a lectin domain

An overview of soluble proteins with a lectin domain involved in plant defense signaling is given in **Table 3**.

***Amaranthins.*** The Amaranthin family groups all proteins related to amaranthin, a lectin present in the seeds of *Amaranthus caudatus*. Native amaranthin is a homodimeric protein built of two 33 kDa subunits, each comprising two tandem-arrayed homologous amaranthin domains. Amaranthin specifically recognizes the T-antigen disaccharide Galβ(1,3)GalNAc but also interacts with GalNAc. Interestingly, the amaranthin domain itself possesses no sugar binding site(s), but the specific head-to-tail arrangement of two amaranthin subunits is necessary to establish the T-antigen disaccharide binding site (Van Damme et al., 2008). Up till now, only amaranthins originating from *Amaranthus* species have been purified and biochemically characterized. This nucleocytoplasmic lectin was reported to enhance the plant’s resistance against aphids when ectopically expressed in transgenic tobacco, potato, and cotton by affecting growth and development of the invading aphids (Wu et al., 2006; Xin et al., 2011).

Screening of the publicly available genome databases revealed that the amaranthin domain is widespread throughout the plant kingdom. Several chimeric proteins containing N-terminal amaranthin domain(s) coupled to unrelated protein domains have been identified (Van Damme et al., 2011). Columbine plants (*Aquilegia formosa*× *Aquilegia pubescens*) encode a protein in which two amaranthin domains are coupled to a kinase domain. Since this protein does not have a transmembrane domain, it is suggested to reside inside the cell. Cucumber, maize, and wheat plants were found to contain genes that encode proteins with amaranthin domain(s) coupled to an aerolysin domain. Aerolysins are cytolytic toxins that are mostly produced by the bacterium *Aeromonas* and can kill host cells upon pore formation in the plasma membrane (Degiacomi et al., 2013). In wheat, a chimeric protein called Hfr-2 (Hessian fly responsive-2) is up-regulated in the leaf sheaths after feeding of virulent Hessian fly larvae and armyworms, and enhances wheat tolerance against Hessian fly larvae (Puthoff et al., 2005).

***Calreticulin/calnexin.*** Calreticulin (CRT) and calnexin (CNX) are glucose binding lectins residing in the endoplasmic reticulum (ER) of eukaryotic cells. Both CRT and CNX act as molecular chaperones and are essential ER components ensuring proper folding and quality control of newly synthesized secretory and membrane-bound glycoproteins before ER release (Ellgaard and Frickel, 2003; Kapoor et al., 2004; Williams, 2006). While CNX is a type-I integral membrane protein, CRT is a soluble protein. Both CRT and CNX act together with their co-chaperones ERp57 and PDI, two soluble thiol-disulfide oxidoreductases. Whereas the classical chaperones associate with the peptide moiety of their substrates, CNX and CRT bind to their glycoprotein substrates primarily through specific recognition and binding to the oligosaccharide intermediates Glc_1_Man_7-9_GlcNAc_2_ present on nascent glycoproteins.

In the ER quality control system, a growing polypeptide initially gets N-glycosylated with the core glycan Glc_3_Man_9_GlcNAc_2_. By successive action of glucosidase I and II, the outer glucoses are trimmed resulting in a monoglucosylated glycoprotein which then serves as the substrate of CNX/CRT for proper folding. Once the glycoprotein is correctly folded, the terminal glucose of its oligosaccharide is cleaved by glucosidase II and the glycoprotein is released from the CNX/CRT/ERp57 complex for further processing. If the glycoprotein is not correctly folded, it is recognized by the UDP-glucose:glycoprotein glucosyltransferase enzyme, which acts as a folding sensor, and gets re-glucosylated to promote its renewed association with CNX/CRT. As such, de- and re-glucosylation of glycoproteins facilitates their correct folding. When folding ultimately fails, the misfolded glycoproteins are sorted out of the ER and are degraded by the proteasome, a system known as ER associated degradation or ERAD. Under adverse environmental conditions, the demand for protein folding exceeds the capacity of the system resulting in the accumulation of misfolded proteins in the ER, giving rise to so-called ER stress (Howell, 2013; Liu and Li, 2014).

The correct folding of membrane-bound PRRs is a critical step in plant immunity. The ER quality control system not only regulates the abundance and quality of transmembrane receptors, it also affects downstream signaling of the receptor (Tintor and Saijo, 2014). The LRR-RLKs *At*EFR and *At*FLS2 from *A. thaliana* as well as the LysM LecRK NFP from *M. truncatula* require proper *N*-glycosylation for accurate functioning. Nevertheless, *At*EFR and *At*FLS2 production are coordinated by different ER components (Li et al., 2009; Häweker et al., 2010). *Arabidopsis* plants contain two types of CRTs: CRT1/2 and CRT3 isoforms (Thelin et al., 2011). Whereas CRT1 is a key chaperone in plant ER stress, CRT3 is typically involved in the quality control of *At*EFR and the brassinosteroid receptor BRI1, but is not essential for *At*FLS2 biogenesis (Jin et al., 2009). CRT2 appears to have a dual regulatory role in plant defense against the biotrophic pathogen *P. syringae* pv tomato DC3000 (Qiu et al., 2012). Upon pathogen invasion, CRT2 is involved in the up-regulation of SA-dependent immune signaling through its Ca^2+^ buffering capacity. However, CRT2 negatively influences these SA-dependent responses through its chaperone activity, resulting in the overall suppression of plant resistance toward *P. syringae* pv tomato DC3000.

***EUL-related lectins.*** The family of EUL-related lectins groups all nucleocytoplasmic proteins that comprise at least one *Euonymus* lectin (EUL) domain. The prototype of this family is the so-called *Euonymus europaeus* agglutinin (EEA) which is present at very high concentrations in the arillus tissue of the spindle tree (*E. europaeus*). EEA is a non-glycosylated homodimeric protein composed of 17 kDa subunits, and recognizes two structurally different classes of glycans. Glycans with carbohydrate epitopes containing galactose, such as Galα1–3Gal and Galα1–3Galβ1–4GlcNAc, blood group B [Galα1–3(Fucα1-2)Gal-], and O (Fucα1–2Gal-) epitopes are bound with a higher affinity compared to high-mannose *N*-glycans. Based on inhibition studies, it was suggested that the EUL domain might contain two different binding sites (Fouquaert et al., 2008).

Sequences with an EUL domain are present in almost all sequenced plant genomes from Embryophyta, ranging from liverworts to flowering plants. The sequence of the EUL domain is well conserved suggesting that the corresponding EUL proteins fulfill an essential role in plants. Based on the overall protein domain architecture, the EUL family can be divided into two groups containing proteins either composed of a single EUL domain (S-type EUL proteins) or of two tandemly arrayed EUL domains separated by a spacer sequence (D-type EUL proteins; Fouquaert and Van Damme, 2012). The majority of the EUL sequences encode chimeric proteins, in which the EUL domain is linked to other unknown domains. Whereas most dicot species encode one or two EUL S-type proteins, monocot, and lower plant species contain a whole set of S- and D-type EUL sequences.

In contrast to EEA, which is expressed at high concentrations in the arilli of spindle tree seeds, the EUL proteins from *Oryza sativa* and *A. thaliana* are very low abundant proteins. In both plants the amount of EUL transcripts is enhanced after the plant was subjected to different abiotic (such as dehydration, salinity, osmotic stress, and ABA treatment) and biotic (such as bacterial and fungal infection) stresses (Fouquaert and Van Damme, 2012; Al Atalah et al., 2014a).

Next to the differential regulation of gene expression, the EUL proteins show different carbohydrate binding properties. ArathEULS3 from *A. thaliana* preferentially interacts with *N*-glycans containing galactosylated structures such as Lewis X [Galβ1–4(Fucα1–3)GlcNAc], Lewis Y [Fucα1–2Galβ1–4(Fucα1–3)GlcNAc] and lactosamine (Galβ1–4GlcNAc) motifs (Van Hove et al., 2011). Similarly, both EUL domains composing the two-domain EUL protein from rice, OrysaEULD1A, show carbohydrate specificity toward galactose containing glycans (Al Atalah et al., 2014b). In contrast, the rice protein OrysaEULS2 preferably binds mannosylated N-glycan structures (Al Atalah et al., 2012). All these EUL proteins are located in the nucleus and the cytoplasm of the plant cell. In search for interacting proteins, Li et al. (2014) recently reported that ArathEULS3 interacts with the nuclear/cytosolic ABA receptor RCAR1. Furthermore, Berendzen et al. (2012) demonstrated interaction of ArathEULS3 with CPK3, a Ca^2+^ dependent kinase involved in the ABA response in guard cells, supporting a role for ArathEULS3 in ABA signaling and stomatal closure.

***Jacalin-related lectins (JRL).*** The family of jacalin-related lectins is named after jacalin, a 18 kDa T-antigen disaccharide-binding lectin domain first isolated from the seeds of jackfruit (*Artocarpus integrifolia*). Based on differences in molecular structure, subcellular localization, and carbohydrate binding properties, the large group of jacalins can be subdivided into two subgroups, further referred to as the galactose binding and mannose binding JRLs, residing in the vacuolar and nucleocytoplasmic compartment of the plant cell, respectively.

Galactose-specific JRLs have been reported mainly within the family Moraceae, whereas the mannose-specific JRLs are widespread in Viridiplantae. Furthermore, recent studies have shown that the jacalin domain is not restricted to plant proteins, since a similar domain has been reported in eukaryotes outside the plant kingdom as well as in some prokaryotes (Van Damme et al., 2008; Kanagawa et al., 2014). In plants, chimeric proteins comprising one or more jacalin domains linked to an unrelated domain are widely distributed. For instance, in *A. thaliana*, sequences composed of one or two jacalin domains C-terminally linked to five in tandem arranged Kelch domains are present. In addition, multiple *Arabidopsis* genes encode a putative F-box protein with a C-terminal jacalin domain (Nagano et al., 2008). Several Poaceae species (wheat, rice, maize) express proteins consisting of an N-terminal dirigent domain (also called disease-response domain) C-terminally fused to a jacalin domain. In rice, additional types of chimerolectins were identified, either composed of an N-terminal tyrosine kinase domain coupled to two or three jacalin domains or an N-terminal NB-ARC motif coupled to a LRR and a C-terminal jacalin domain (Van Damme et al., 2008).

Many jacalin-related lectin genes have been shown to be associated with disease resistance, abiotic stress signaling, wounding, insect damage or multiple stresses (Song et al., 2014). Especially the jacalin proteins with a dirigent domain are functionally involved in plant defense. To our knowledge, these chimeric proteins have only been reported in Poaceae species. In wheat, nearly half of the jacalin-related lectin genes encode dirigent domain-containing jacalin-related proteins. Several of these proteins have been studied in some detail, amongst them TaVER2, TaHfr-1, and TaJA-1 (Song et al., 2014). Interestingly all these mannose binding lectins are expressed as a response toward plant stress. TaVER2 is specifically expressed during vernalization in wheat (Yong et al., 2003) but is also up-regulated upon jasmonate and ABA treatment (Feng et al., 2009). TaHfr-1 is up-regulated after herbivory of Hessian fly larvae (Williams et al., 2002) and TaJA-1 is specifically accumulating after jasmonate treatment (Ma et al., 2013). The mannose specific TaHfr-1 was shown to effectively inhibit Hessian fly larval feeding resulting in the delay of larval development and premature death of the pest insects (Subramanyam et al., 2008). Transgenic tobacco plants overexpressing Ta-JA1 revealed increased resistance to bacterial, fungal, and viral pathogens (Ma et al., 2010). TaVER2 homologs have been found in maize and sorghum (β-glucosidase aggregating factor) and in rice (OsJAC1; Esen and Blanchard, 2000; Jiang et al., 2006; Kittur et al., 2009). Transgenic rice plants overexpressing OsJAC1 indicated the importance of OsJAC1 for rice growth and development (Jiang et al., 2007).

Similar to the chimerolectins also jacalin-related proteins composed only of jacalin domains are up-regulated in plant tissues subjected to certain stress treatments. For instance, Orysata was first reported as a salt inducible mannose binding lectin in the leaves of *O. sativa* (Zhang et al., 2000). Later it was shown that Orysata is also expressed upon JA and ABA treatment, after infection with an incompatible *Magnaporthe grisea* strain as well as during senescence (Lee et al., 2001; de Souza Filho et al., 2003; Qin et al., 2003). Glycan array analyses revealed that Orysata preferentially interacts with high-mannose and some more complex *N*-glycans (Al Atalah et al., 2011). In recent years, several orthologs of Orysata have been identified in Gramineae species but also in other plants, such as *Helianthus tuberosus* and *Ipomoea batatas*. The mannose specific wheat protein TaJRLL1 consisting of two jacalin-like domains is considered a component of SA and JA dependent plant defense signaling mechanisms and is activated upon fungal infection (*Fusarium graminearum* and *Blumeria graminis*; Xiang et al., 2011). However, not all jacalin-related defense proteins depend on hormone signaling. For example, the *Arabidopsis* jacalin-related JAX1 confers broad but specific resistance to potex viruses by inhibition of viral RNA accumulation, independent of hormone signaling (Yamaji et al., 2012). Other *Arabidopsis* jacalin-related proteins interact with proteins of ER bodies, i.e., ER-derived organelles presumably involved in defense against herbivores and/or pathogens (Nagano et al., 2008). Recently, the jacalin-related protein from sunflower seedlings named Helja was reported as a lectin with antifungal properties toward some pathogenic fungi of the genus *Candida*. Helja induces morphological changes as well as ROS production in yeast cells. Furthermore, lectin treatment also altered the membrane permeability of the cells (Regente et al., 2014).

***Nictaba-related lectins.*** The family of Nictaba-related lectins was named after the *Nicotiana tabacum* agglutinin, abbreviated as Nictaba, a 19 kDa lectin domain originally discovered in tobacco leaves (Chen et al., 2002a) after jasmonate treatment. Though the lectin was first reported as a chito-oligosaccharide binding protein, glycan array analyses revealed that Nictaba also reacts with the inner core structure (Man)_3_β1–4GlcNAcβ1–4GlcNAcβ-N-Asn of high-mannose and complex *N*-glycans. Biochemical assays confirmed that Nictaba can interact in a sugar-inhibitable way with many N-glycosylated proteins (Lannoo et al., 2006, 2007). A nuclear proteomics approach revealed the interaction of Nictaba with the core histone proteins H2A, H2B, and H4 through their *O*-GlcNAc modification (Schouppe et al., 2011), which was later confirmed at the microscopical level (Delporte et al., 2014a).

An extensive survey of the genome/transcriptome databases indicated that Nictaba-like domains are widespread among the Embryophyta but are absent from other eukaryotes and prokaryotes. Few proteins belonging to the Nictaba-like family consist of a single Nictaba domain. Furthermore, numerous sequences were identified encoding chimeric proteins comprising the Nictaba domain fused to unrelated N-terminal domains (e.g., F-box domain) or a Nictaba domain C-terminally fused to an N-terminal TIR (toll/interleukin-1 receptor) domain (found in *Arabidopsis* and tomato), an AIG1 (avrRpt2-induced gene) domain (identified in *Arabidopsis*) or a kinase domain (found in rice; Delporte et al., 2014b).

At present, only few Nictaba-related proteins have been studied for their biological properties and physiological role. A comparative analysis of the carbohydrate binding properties of Nictaba from *N. tabacum*, the Cucurbitaceae phloem lectin PPL, the *A. thaliana* homolog PP2-A1 and the *A. thaliana* F-box-Nictaba protein encoded by the gene *At2g02360* revealed that despite the sequence similarity and the presence of conserved amino acids in the carbohydrate binding site, different Nictaba domains can interact with different glycan motifs (Delporte et al., 2014b), suggesting different biological roles.

Since insect herbivory by Lepidopteran insects also triggers the JA pathway the tobacco lectin also accumulates after caterpillar attack. Furthermore, feeding assays with (transgenic) tobacco lines demonstrated the entomotoxic activity of Nictaba. It was suggested that the entomotoxic effect of Nictaba is caused by interaction of the lectin with glycoconjugates present in the digestive tract of the insect (Vandenborre et al., 2010, 2011). Within the plant cell, insect herbivory results in enhanced Nictaba expression in the cytoplasm, followed by partial translocation of the lectin to the nucleus, where it can interact with core histone proteins through their *O*-GlcNAc modification. It is hypothesized that Nictaba binding to chromatin results in enhanced transcription of defense related genes (Lannoo and Van Damme, 2010).

The Cucurbitaceae phloem lectins are a group of Nictaba-related lectins that are found in phloem exudates of different Cucurbitaceae species. Unlike Nictaba, the PP2 lectins are exclusively and continuously expressed in the companion cells of the phloem and then translocated into the phloem sap. The pumpkin lectin PPL and the PP2-like protein from *A. thaliana*, PP2-A1, show high binding affinity for chitin oligomers (GlcNAc_3-6_). Similar to Nictaba, PP2-A1 also binds with the Man_3_GlcNAc_2_ core of high-mannose *N*-glycans (Beneteau et al., 2010). Interestingly, the expression of PP2-A1 was enhanced by ethylene treatment and *Pseudomonas* infection. Furthermore, PP2-A1 represses phloem feeding of the green peach aphid *Myzus persicae* (Zhang et al., 2011), displayed antifungal activity against various fungal strains (Lee et al., 2014), and negatively affects Cucurbit aphid borne yellow virus transmission (Bencharki et al., 2010), strongly supporting a role in plant defense.

Several F-box Nictaba proteins are encoded in the *A. thaliana* genome. Glycan array analyses revealed the F-box protein encoded by *At2g02360* exhibits carbohydrate binding activity toward *N*- and *O*-glycans with *N*-acetyllactosamine (LacNAc; Galβ1–3GlcNAc and Galβ1–4GlcNAc) and poly-LacNAc structures as well as with Lewis A (Galβ1–3(Fucα1–4)GlcNAc), Lewis X (Galβ1–4(Fuca1–3)GlcNAc), Lewis Y (Fucα1–2Galβ1–4(Fucα1–3)GlcNAc), and type-1 B motifs (Galα1–3(Fucα1–2)Galβ1–3GlcNAc. Since these glycan structures have been reported in bacteria, viruses, and animals rather than in plants the physiological importance of this glycan interaction remains enigmatic (Lannoo et al., 2008; Stefanowicz et al., 2012). Furthermore, the same *Arabidopsis* F-box Nictaba protein was shown to interact with core members of an E3-type ubiquitin ligase complex which resulted in the hypothesis assuming a role of the Nictaba domain in glycoprotein degradation (Takahashi et al., 2004; Arabidopsis Interactome Mapping Consortium, 2011).

***Ricin-B lectins.*** The ricin-B lectin family is one of the most widespread families of carbohydrate binding proteins in nature. The most famous member of this family is ricin, a toxic protein from castor bean (*Ricinus communis* L.) seeds, which was the very first lectin discovered in plants by Peter Hermann Stillmark in 1888. Ricin is a chimeric protein consisting of an A chain with enzymatic activity linked through a disulfide bridge with a B chain with lectin activity. This B chain consists of a duplicated ricin-B domain, responsible for the carbohydrate binding activity of the protein toward galactosylated structures. The enzymatic activity of ricin involves RNA *N*-glycosidase activity and is responsible for the removal of a highly conserved adenine residue from the sarcin/ricin loop of the 28S ribosomal RNA. As a result, the ribosomes are no longer able to bind elongation factor 2 and protein synthesis is blocked. Because of their catalytic activity these chimeric proteins are also referred to as type 2 ribosome-inactivating proteins (RIPs), and are considered as toxic proteins if they succeed in entering the cell. The uptake of the protein by the host cell is aided by their lectinic B-chain which can specifically interact with glycoconjugate structures on the cell surface. Except for ricin and abrin (from the jequirity bean *Abrus precatorius*), most type 2 RIPs are only moderately or even weakly toxic (Van Damme et al., 2001; Stirpe and Battelli, 2006).

The family of ricin-B related lectins is widespread in the plant kingdom and has been characterized in detail for what concerns its biological activity and toxicity in several plant species, especially *R. communis* (castor bean), *Abrus precatorius* (jequirity bean), *Viscum album* (mistletoe), and *Sambucus nigra* (elderberry). Unlike the other soluble lectins described above, most ricin-B related proteins accumulate in the plant vacuole or are secreted to the extracellular space (Van Damme et al., 2008). Over the years the ricin-B domain was identified in numerous plants, animals, fungi, and bacteria. All these proteins with ricin-B domains are also classified as the R-type lectins (Cummings and Etzler, 2009).

Within the genus *Sambucus* (elderberry) an extended family of ricin-B related proteins, including several chimerolectins and hololectins has been identified (Shang and Van Damme, 2014). Detailed hapten inhibition assays and glycan array studies revealed that all these *S. nigra* proteins exhibit different carbohydrate binding properties, and allowed to classify the *Sambucus* lectins into three groups. A first group covers the lectins SNA-II and SNA-IV as well as the type 2 RIP SNA-V with specificity toward Gal/GalNAc and Gal/GalNAc-containing glycan structures. The second group comprises only the type 2 RIP SNA-I, which specifically interacts with terminal sialic acid residues (Neu5Ac; α2–6) linked to Gal/GalNAc. Finally, the type 2 RIP SNLRP represents a third specificity group with strong interaction with GlcNAc oligomers (Shang and Van Damme, 2014).

Several lines of evidence support the idea that ricin-B related lectins play a role in plant defense against pathogens (Chen et al., 2002b; Vandenbussche et al., 2004a,b) and insects (Wei et al., 2004; Shahidi-Noghabi et al., 2009). Over-expressing SNA-I′ or SNA-V from *S. nigra* in transgenic tobacco (Samsun NN) plants enhances the tobacco plant’s resistance against infection with tobacco mosaic virus. Though the antiviral effect is clearly related to the amount of protein expressed it cannot be related to an increased expression of pathogenesis-related proteins (Chen et al., 2002b; Vandenbussche et al., 2004a,b). Furthermore, no clear correlation was observed between *in planta* antiviral activity of the transgenic tobacco lines and the *in vitro* *N*-glycosidase activity of the proteins toward genomic RNA of the tobacco mosaic virus, suggesting that the *in planta* antiviral activity of these RIPs may rely on a direct interaction with the virus (Vandenbussche et al., 2004a). At present the importance of the lectin activity in the antiviral activity of the proteins remains unclear.

The first evidence for the insecticidal activity of ricin-B related proteins came from feeding assays with ricin and cinnamomin (from *Cinnamomum camphora* tree). Ricin showed strong toxicity to several insects such as cowpea weevil (*Callosobruchus macultatus*), cotton boll weevil (*Anthonomus grandis*), house fly (*Musca domestica*), and larvae of the silkworm *Bombyx mori* (Wei et al., 2004). The differences in activity between ricin and cinnamomin could not be related to the enzymatic activity but rather were attributed to differences in the activity of the lectin chain of the proteins (Wei et al., 2004). Shahidi-Noghabi et al. (2009) reported the enhanced resistance of transgenic tobacco plants overexpressing SNA-I or its isoform SNA-I′ toward different pest insect species including aphids and caterpillars. Since mutation of the carbohydrate binding site can abolish or reduce the toxic effect, the entomotoxic properties of the proteins can be linked to their carbohydrate binding activity (Shahidi-Noghabi et al., 2008). In addition, the cytotoxic effects of *S. nigra* RIPs toward insect cells was accompanied by caspase 3-like protease-induced apoptosis (Shahidi-Noghabi et al., 2008, 2011). More research is needed to identify the interacting proteins for the *Sambucus* lectins on the cell surface.

## CONCLUSION

Plant genomes encode a plethora of RLKs, RLPs, and lectins to protect themselves against the vast array of pathogenic bacteria, viruses, fungi, oomycetes, and pest insects. A key feature of the plant’s innate immunity is the ability to recognize D/P/MAMPs of potential pathogens through PRRs, and subsequently respond in a highly sensitive and specific manner. Many advances have been made in the understanding how different proteins function in plant innate immunity. As more structural and biochemical data become available, common themes are emerging on receptor organization, ligand perception and binding, receptor activation, and intracellular defense signaling. It is clear now that PRRs are ultradynamic multiprotein structures which often use phosphorylation to activate downstream signaling.

A first interaction between the pathogen and the plant occurs at the level of the cell wall and the plasma membrane where extracellular effectors, DAMPs, and P/MAMPs will be recognized by membrane-bound receptors (**Figure 1**; ), among which a large group of RLPs and RLKs some of which carry an extracellular lectin domain. Though different lectin motifs have been recognized, only the LysM domain was unambiguously shown to be dependent on carbohydrate interactions for recognition of fungal and bacterial components and subsequent signal transmission into the plant cell. Interestingly, the LysM motif shows high specificity for chito-oligosaccharides, an abundant component in different pathogens but absent from plants. Other (lectin) receptor kinases/proteins probably depend on protein–protein interactions to recognize specific ligands. Though our knowledge on receptor kinase function and signaling has greatly improved, several issues still remain with respect to the potential ligands for pathogen recognition and the downstream signaling events, especially for those receptors lacking the kinase domain.

In addition to the lectin motifs present at the level of the cell wall/plasma membrane, plants synthesize well-defined soluble lectins upon exposure to multiple abiotic and biotic stresses (**Table 3**). Most of these inducible lectins reside in the nucleus and the cytosol of the plant cell and evidence is emerging for their role in signal transduction inside the plant cell as part of multiple plant defense pathways. Hence protein–carbohydrate interactions should not only be envisaged at the level of the interaction between the pathogen and the plant cell, but also play an important role inside the plant cell as part of the intracellular signaling resulting from the recognition of plant attackers. At least for some cytoplasmic lectins (Nictaba-related proteins, EUL-related proteins, amaranthins) it was shown that they are also translocated inside the plant nucleus. The Nictaba-related proteins in particular have been shown to interact with glycosylated histones, and therefore are suggested to act as chromatin remodelers, enabling altered gene expression as a result of stress signaling. Surveying the plant genome sequences also revealed that most lectin domains are part of larger proteins, consisting of one or more lectin domains linked to un-related protein domains, most often with unknown functions. Future challenges include the characterization of the ligands for these soluble lectins or lectin domains present as part of a larger protein to elucidate the biological relevance of these interactions. Large-scale experiments integrating genomics, biochemistry, cell biology, structural biology, and bioinformatics will enable to elucidate the physiological importance of the lectin motifs in protein–carbohydrate interactions in signal transduction and plant defense.

## Conflict of Interest Statement

The authors declare that the research was conducted in the absence of any commercial or financial relationships that could be construed as a potential conflict of interest.
